# Dissecting the Role of AXL in Cancer Immune Escape and Resistance to Immune Checkpoint Inhibition

**DOI:** 10.3389/fimmu.2022.869676

**Published:** 2022-04-27

**Authors:** Agnete S. T. Engelsen, Maria L. Lotsberg, Raefa Abou Khouzam, Jean-Paul Thiery, James B. Lorens, Salem Chouaib, Stéphane Terry

**Affiliations:** ^1^Centre for Cancer Biomarkers and Department of Biomedicine, University of Bergen, Bergen, Norway; ^2^Thumbay Research Institute for Precision Medicine, Gulf Medical University, Ajman, United Arab Emirates; ^3^Guangzhou Laboratory, Guangzhou, China; ^4^Inserm, UMR 1186, Integrative Tumor Immunology and Immunotherapy, Villejuif, France; ^5^Gustave Roussy, Villejuif, France; ^6^Faculty of Medicine, University Paris Sud, Le Kremlin-Bicêtre, France; ^7^Research Department, Inovarion, Paris, France

**Keywords:** AXL, TAM receptors, EMT, cell plasticity, tumor microenvironment, immunotherapy, immune evasion, immunosuppression

## Abstract

The development and implementation of Immune Checkpoint Inhibitors (ICI) in clinical oncology have significantly improved the survival of a subset of cancer patients with metastatic disease previously considered uniformly lethal. However, the low response rates and the low number of patients with durable clinical responses remain major concerns and underscore the limited understanding of mechanisms regulating anti-tumor immunity and tumor immune resistance. There is an urgent unmet need for novel approaches to enhance the efficacy of ICI in the clinic, and for predictive tools that can accurately predict ICI responders based on the composition of their tumor microenvironment. The receptor tyrosine kinase (RTK) AXL has been associated with poor prognosis in numerous malignancies and the emergence of therapy resistance. AXL is a member of the TYRO3-AXL-MERTK (TAM) kinase family. Upon binding to its ligand GAS6, AXL regulates cell signaling cascades and cellular communication between various components of the tumor microenvironment, including cancer cells, endothelial cells, and immune cells. Converging evidence points to AXL as an attractive molecular target to overcome therapy resistance and immunosuppression, supported by the potential of AXL inhibitors to improve ICI efficacy. Here, we review the current literature on the prominent role of AXL in regulating cancer progression, with particular attention to its effects on anti-tumor immune response and resistance to ICI. We discuss future directions with the aim to understand better the complex role of AXL and TAM receptors in cancer and the potential value of this knowledge and targeted inhibition for the benefit of cancer patients.

## 1 Introduction

Receptor tyrosine kinases (RTKs) are classified into families based on similarities in their amino acid sequence, structural and functional properties. The TAM (TYRO3, AXL, MERTK) receptor family is characterized by an intracellular kinase domain and unique extracellular domains containing pairs of immunoglobulin (Ig)-like and fibronectin type III (FNIII) domains. AXL (also known as UFO, TYRO7, ARK) was the first cloned member of the TAM receptors (1). *AXL* was characterized as a novel transforming gene in chronic myeloid leukemia cells in 1991 (1, 2), and subsequent cloning of *TYRO3* (3) and *MERTK* in 1994 revealed their structural and functional similarities (4). These receptors have critical physiological functions in innate immunity, central nervous system development, angiogenesis, and platelet aggregation (5, 6). Deregulation of TAM receptors has been linked to the pathogenesis of numerous human diseases, including cancer (6–9). Due to their proposed role in tumor promotion, the TAM receptor family has received considerable attention over the past decade (10).

Accumulating evidence reveals a multifaceted role of AXL in promoting immunosuppression and resistance to anti-tumor immunity. To escape anti-tumor immunity, cancer cells exploit cell-intrinsic pathways associated with resistance to immune cell-mediated attack and avoid recognition by anti-tumor immune cell types (11–17). The cancer cells may also enhance immunosuppression of the tumor microenvironment (TME), and specifically the tumor immune microenvironment (TIME), regulating the expression or secretion of immunosuppressive molecules including cytokines and chemokines. This intercellular communication system allows effective inhibition of immune effector cells including T-cells, natural killer (NK) cells, and dendritic cells (DCs) while promoting the functions and/or the recruitment of immunosuppressive cell populations such as regulatory T cells (Tregs), tumor-associated macrophages, and myeloid-derived suppressor cells (MDSCs) (18–20). In this review, we aim to summarize the current knowledge regarding the regulation and function of AXL in cancer and discuss mechanisms involving AXL in the escape from anti-tumor immunity. We also present emerging strategies to target AXL or TAM receptors to improve immunotherapy efficacy.

## 2 The Biology of TAMs and GAS6/AXL Signaling

TAM receptors are expressed by various cell types and activated by vitamin K-dependent ligands, growth arrest-specific factor 6 (GAS6), and protein S (PROS1), representing the two best-characterized TAM ligands. TYRO3, AXL, and MERTK exert multiple functions, and despite the partial overlap, the three TAM receptors display different expression patterns (8, 9, 21, 22). The TAM receptors also exhibit distinct activation patterns. PROS1 binds to MERTK and TYRO3 with the highest affinity, whereas GAS6 can bind to all three TAM receptors with the highest affinity for AXL. Other putative ligands have been proposed to activate MERTK and/or TYRO3, such as Tubby (23) and galectin-3 (24). Genetic deletion of all three TAM receptors (*Tyro3/Axl/Mertk* triple knock-out in mice) is not embryonically lethal. However, the animals display various postnatal phenotypes associated with thromboembolic disease, atherosclerosis, sepsis, inflammatory and autoimmune diseases (25). These phenotypes may be partly explained by a reduced ability to clear apoptotic cells observed in several tissues in association with increased levels of pro-inflammatory cytokines (e.g., TNF-α, IL-6) (25, 26). Indeed, TAM receptors are expressed on phagocytic cells, and alteration of TAM-mediated phagocytosis perturbs the clearance of apoptotic cells. Loss of AXL has further been shown to increase neuroinflammation (27) and vascular permeability (28).

**Figure 1:**
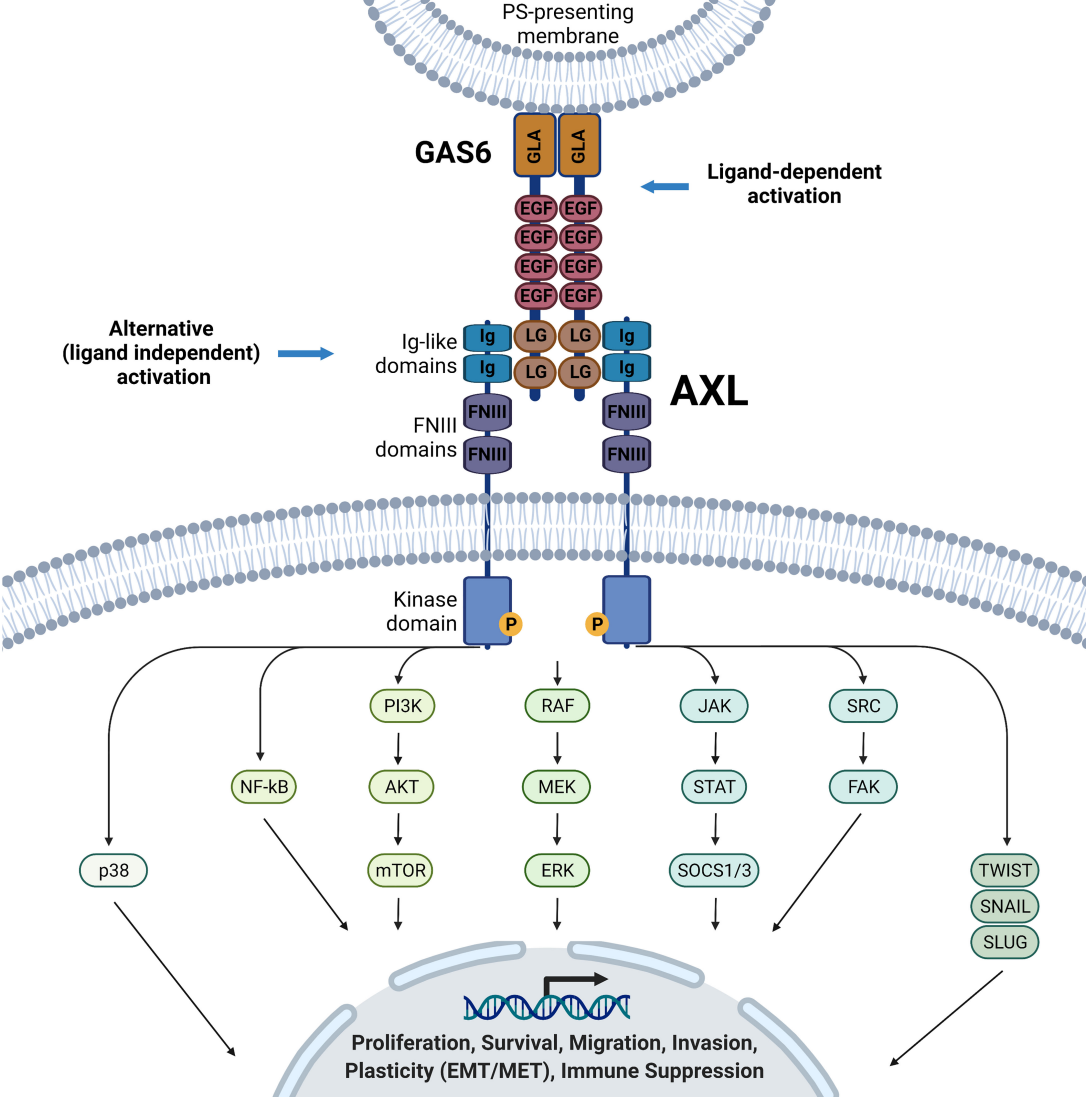
GA6/AXL structure and downstream signaling pathways. Ligand binding of the AXL receptor tyrosine kinase promotes autophosphorylation and activates various downstream signaling pathways in a cell- and context-dependent matter, including, but not restricted to p38, NF-kB, PI3K/AKT/mTOR, RAF/MEK/ERK, JAK/STAT/SOCS1/3, SRC/FAK, TWIST, SNAIL and SLUG signaling pathways. These pathways will lead to multiple phenotypes, including proliferation, survival, migration, plasticity, and immune suppression. Ligand-independent mechanisms of AXL activation have been proposed but are not detailed here.

Canonical activation of AXL signaling requires its ligand GAS6 (**Figure 1**) (29, 30). The 678-amino acid GAS6 protein contains a gamma-carboxyglutamic acid (GLA) domain in the N-terminus, essential for binding externalized phosphatidylserine on plasma membranes, four epidermal growth factor (EGF)-like repeats, and tandem globular (G) domains in C-terminus [also referred to as sex hormone-binding globulin (SHBG) or laminin G-like (LG)]. The latter enables GAS6 binding to the Ig-like domains of AXL (**Figure 1**). Moreover, the vitamin K-dependent γ-carboxylation of the TAM ligand GLA domains is essential to elicit full activation of TAMs (31, 32). On the other hand, the accumulation of soluble Ig-like TAM domains with antagonist activity can act as a regulatory mechanism of GAS6/AXL signaling (33, 34). As revealed by structural analysis, GAS6-AXL complexes can assemble into a complex with 2:2 stoichiometry, likely leading to AXL dimerization and activation *via* trans-autophosphorylation of tyrosine residues of the intracellular domain (35). Important tyrosine residues in the intracellular domain include the activation loop (Tyr698, Tyr702, Tyr703) and the C-terminal domain (Tyr779, Tyr821, Tyr866), which are necessary for the recruitment of adaptor proteins mediating signaling cascades including the adaptor GRB2 leading to the activation of phosphatidylinositol 3 kinase (PI3K), phospholipase C (PLC), or SRC kinase. In a cell type- and tissue-dependent context, it triggers the downstream activation of various signaling pathways, including PI3K-AKT, NF-KappaB; RAS-MEK-ERK, JAK-STAT, SRC/FAK (6, 7, 21, 22, 36–38). In addition to the canonical GAS6/AXL activation pathway, evidence is accumulating that malignant cells have developed various ways to bypass, at least in part, their dependence on GAS6 (21, 39–41). Different AXL-mediated signaling pathways may support cell-autonomous or cell-to-cell mediated crosstalk during cancer progression. AXL overexpression and heterodimerization with non-TAM RTK members such as MET- (42–44), epidermal growth factor receptor- (EGFR) (43, 45–47), HER2- (44, 48) and HER3- (49, 50) mediated transactivation of AXL have been reported in various cancer systems. AXL’s interaction or oligomerization may also cause accumulation of AXL at the cell surface (44, 48). In some cases, this crosstalk could diversify RTK signaling in the cancer cells in a ligand-dependent or independent manner (43, 44). In ovarian carcinoma cells, upon GAS6 activation, AXL was reported to co-cluster with and transactivated MET, EGFR, and HER2, resulting in downstream activation of ERK (44). Additionally, the tumor suppressor OPCML was found to interact and promote AXL inactivation in cholesterol-rich, detergent insoluble membrane compartments, where proximity to another tumor suppressor, PTPRG phosphatase, resulted in AXL-dephosphorylation, preventing AXL-mediated transactivation of other RTKs (cMET and EGFR) and downstream signaling (51). There is need to further dissect the cooperative regulatory events that may be cancer type specific, heterogenous, and context-dependent, and generalizations should be avoided.

## 3 AXL Expression in Cancer

Although AXL was first isolated and described as a potential oncogene in chronic myeloid leukemia (1, 2), it was later found to be overexpressed in solid tumors and numerous hematological cancers [reviewed in (6, 7, 52–54)]. In contrast to many other RTKs associated with cancer, genetic aberrations of AXL are uncommon. AXL is rather frequently upregulated at the transcriptional level (55). Overexpression of AXL has been correlated with disease aggressiveness in many cancers. This includes cancers of breast (56–58), lung (53, 59–62), gastrointestinal (63–66), head and neck (67–69), hepatocellular carcinoma (70–72), renal cell carcinoma (RCC) (73–77), gynecological carcinoma (78–81), gliomas (82) pancreatic cancer (83, 84), and thyroid carcinoma (85). Overexpression of AXL has also been reported in several types of sarcomas (86–89), in acute myeloid leukemia (AML), and other hematologic malignancies (52, 90, 91). Some studies have reported AXL as an independent prognostic marker (56, 62, 68, 70, 72, 73, 78, 82, 83, 87, 90–92). Moreover, soluble forms of AXL resulting from shedding of the receptor have been characterized, with potential utility for patient monitoring in certain malignancies (73, 93–96).

## 4 The Regulation of AXL Gene Expression

**Figure 2:**
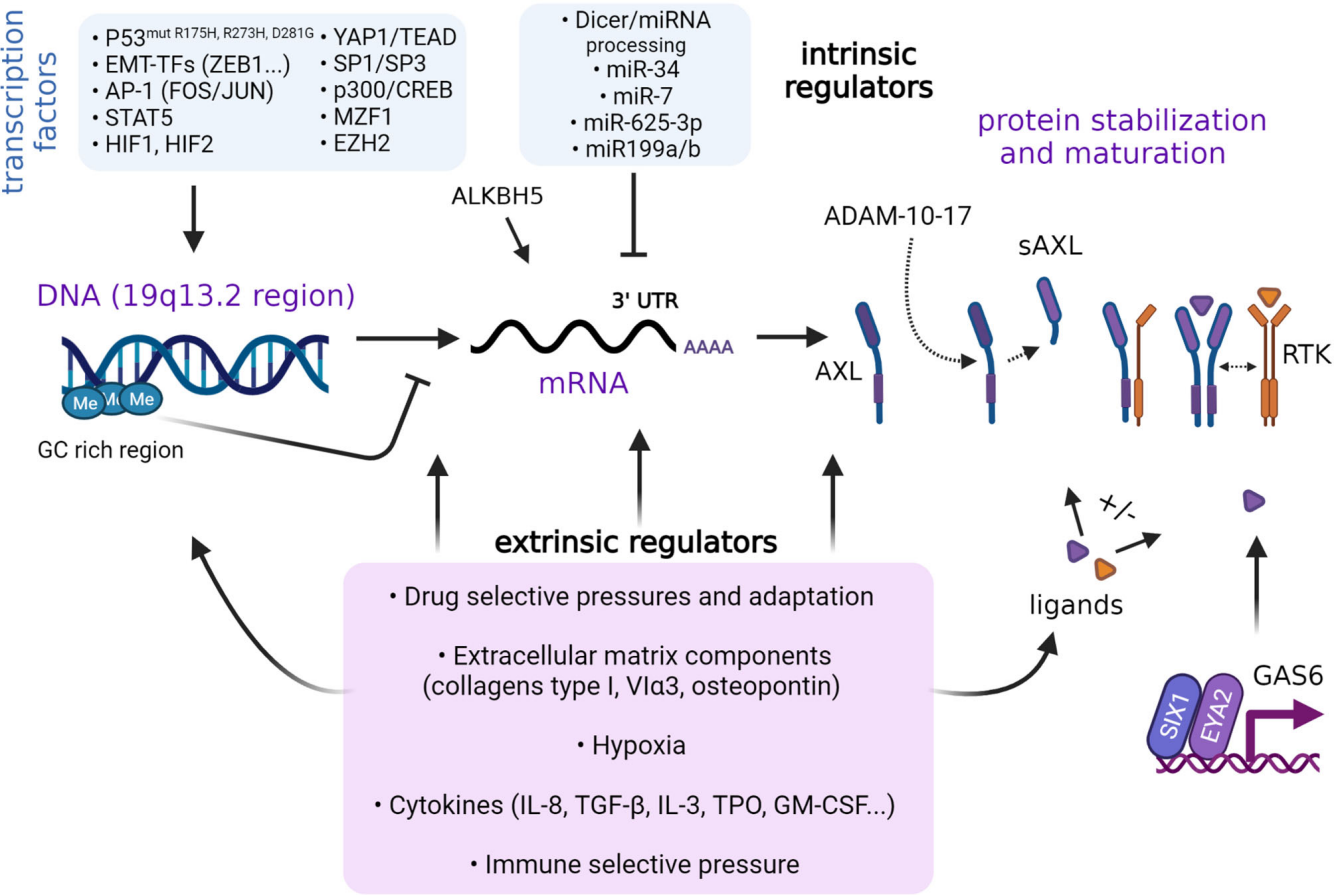
Regulation of AXL expression. Regulation of AXL expression is context-dependent and involves intrinsic and extrinsic factors. Various transcription factors and epigenetic events such as DNA methylation have been identified to regulate AXL expression. AXL protein synthesis is partly regulated by miRNAs. The stabilization of AXL can be affected by ligand binding and interactions with other RTKs. Cleavage of AXL extracellular domain into a soluble form by the action of A Disintegrin And Metalloprotease (ADAM) 7-10. Extrinsic factors, stress, and microenvironmental conditions may also control the different steps. The role of AXL as a sensor of the environmental cues in specific cancer systems and at various stages of cancer progression remains to be fully elucidated.

Many intrinsic factors are known to regulate AXL expression (**Figure 2**). As demonstrated in non-small cell lung cancer (NSCLC) cells, AXL is part of a group of genes controlled by methylation of cytosine nucleotides in their promoter region rich in GC repeats (97). Mudduluru and colleagues identified a GC-rich region (-556 to +7) containing specificity protein (SP)-binding sites sufficient to regulate basal AXL promoter activity in multiple cancer cell lines. Thus, SP1 and SP3 expression levels govern AXL promoter activity. The same investigators also reported on the importance of the Myeloid zinc finger 1 (MZF1) binding to the AXL promoter and regulating its expression in cervical (HeLa) and colorectal cancer (Rko) cells. In AML cells, STAT5 binds and significantly enhances AXL promoter activity following activation by cytokines (98). ALKBH5 is a key positive regulator of AXL mRNA stability in AML cells (99). In leukemia cells, AXL expression is regulated by the activator protein 1 (AP-1), FOS and JUN heterodimers (100). AXL expression can be upregulated through MAPK signaling to JUN. AP-1-mediated regulation of AXL is not restricted to leukemia cells. In NSCLC and head and neck squamous cell carcinoma (HNSCC) cells, JUN overexpression coincides with acquired resistance to cetuximab and is accompanied by increased expression of AXL (101). Similar regulatory effects were observed in cell lines resistant to the PI3K inhibitor BYL719 (alpelisib) (102). Epigenetic modifiers, including EZH2, sustain AXL expression in glioblastoma cells in a manner that seems independent of histone or DNA methylation (103). YES-associated protein 1 (YAP1) is another key co-transcriptional regulator of AXL in various cancer systems. As downstream effector of the Hippo pathway (104), the functions of YAP1 have been attributed to its interaction with the TEAD transcription factor, which can bind TEAD-binding domains present within the AXL promoter to transactivate AXL gene expression (105–107). Further, ZEB1 overexpression can enhance AXL gene transcription, presumably through YAP1/TEAD activation (106).

MicroRNAs such as miR-199a/b, miR-7, and miR-34a (108–110), as well as long non-coding RNAs (111), have been reported to negatively regulate AXL translation in cancer cell lines. Hypoxia-inducible factors HIF-1 and HIF-2 were found to directly bind to hypoxia-response elements in the AXL proximal promoter (112). Hypoxia promotes AXL expression in cancer cells derived from solid and liquid tumors (98, 112). In RCC cells, pseudo-hypoxia owing to Von Hippel-Lindau (VHL) deficiency upregulates AXL expression (112). In NSCLC cell clones exhibiting a mesenchymal phenotype, hypoxia and HIF-1 was shown to mediate maintenance of AXL expression (113). Of note, hypoxia may not only act on transcriptional regulation of AXL but also stabilize GAS6/AXL signaling by preventing GAS6-mediated downregulation of AXL, at least in DU145 and PC3 metastatic prostate cancer cell lines (114). Much remains to be learned about the exact molecular network and sequence of events at play in distinct cell-types and various cancer-dependent contexts.

## 5 AXL and Therapy Resistance

AXL plays a multifaceted role in cancer therapy resistance. In particular, AXL was recognized as a mediator of drug resistance in ovarian cancer cell lines that acquired resistance to cisplatin (115). Since then, AXL has been shown to contribute to resistance against numerous cytotoxic agents (116), radiation (117), and various targeted therapies (101) [reviewed in (21, 37, 55, 118–120)]. In an unbiased analysis of 643 human cancer cell lines, AXL was strongly associated with a drug-resistant mesenchymal phenotype, and inhibition of AXL displayed a specifically synergistic effect together with antimitotic drugs such as docetaxel (121). Furthermore, AXL was shown to play an important role in mediating resistance to EGFR tyrosine kinase inhibitors in NSCLC. Mutations activating EGFR are prevalent in NSCLC, but most patients develop acquired resistance to the EGFR inhibitors (122–126). Second- and third-generation inhibitors have been developed to overcome therapy resistance. Still, acquired resistance also occurs against these latest-generation therapies, and AXL has been implicated in this setting. For example, AXL expression has been associated with adverse clinical outcomes upon treatment with the third-generation EGFR inhibitor Osimertinib, which is also effective against lung carcinoma cells with secondary T790M mutations (49, 127). AXL inhibition was further shown to inhibit the emergence and persistence of cells tolerant to osimertinib treatment (127). Previous studies found that AXL correlated with resistance to other targeted therapies in NSCLC, including therapies directed to ALK (128, 129), PARP (130), and VEGF/VEGFR (131). In many other cancer types, AXL signaling is a common resistance mechanism to targeted therapies, including ERK/MEK inhibitors (132–134), BRAF inhibitors (133–135), imatinib (136), sunitinib (137), WEE1 inhibitors (138), or lapatinib (139). Overexpression or hyperactivity of AXL is frequently observed in cancer in the context of tumor heterogeneity, plasticity, and the development of therapy-resistant persister cell populations.

## 6 AXL and Epithelial-Mesenchymal Plasticity

A novel concept on the prominent role of AXL in cellular plasticity and as a sensor of the microenvironment that favors therapy resistance is emerging. Epithelial cells are characterized by their highly polarized nature and organization into epithelial sheets with prominent intercellular adherence. Epithelial cells organized into sheets by strong anchoring junctions constitute very potent barriers to macromolecules (140, 141). In contrast, mesenchymal cells exhibit spindle-like morphology, with some punctate adhesions associated with migratory and invasive phenotypes. The process by which epithelial cells transform into mesenchymal-like cells is referred to as the epithelial-to-mesenchymal transition (EMT) (140–142). At the molecular level, EMT is characterized by changes in the expression of multiple proteins including downregulation of epithelial markers such as E-cadherin (*CDH1*) and EpCAM (*EPCAM*) and upregulation of mesenchymal markers such as N-cadherin (*CDH2*) and Vimentin (*VIM*). Some of the best-known EMT-associated transcription factors (EMT-aTFs) include SNAIL (*SNAI1*), SLUG (*SNAI2*), Twist family BHLH transcription factor 1 (*TWIST1*), Zinc finger E-box binding homeobox 1 and 2 (*ZEB1* and *ZEB2*). However, many other transcription factors are involved in the regulation of EMT (140–142), and numerous extracellular triggers converge to induce EMT pathways. Notably, the process of EMT may also be reversed by the mesenchymal-to-epithelial transition (MET). EMT is characterized by a continuum, where cells may transit back and forth along the EMT spectrum. This ability is frequently referred to as epithelial-mesenchymal-plasticity (EMP), and serves to generate intermediate EMT phenotypes (also known as intermediate E/M cells or hybrid cells) (143). Cells of a hybrid or intermediate E/M phenotype are prone to acquire stemness properties, and this population is further believed to be an essential source of therapy-resistant persister-cells (144–146). While most human cancers are of epithelial origin, i.e., carcinomas, it is worth noting that mechanisms similar to EMT and epithelial plasticity also play a role in cancers of non-epithelial origin. Under pathological conditions, markers of EMT have been associated with poor prognosis, metastatic spread, and therapy resistance, and this knowledge has paved the way for more effective and durable antitumor treatments (142, 143, 146–148). Epithelial plasticity has recently been proposed as a hallmark of cancer (145, 146, 149).

The AXL receptor has been closely associated with epithelial cell plasticity. In an isogenic human mammary epithelial cell (HMEC) progression series, AXL expression was shown to be upregulated by specific microenvironmental factors together with KIT expression (150). This finding highlighted the molecular link between AXL and increased cell plasticity, and possibly a key regulatory event for the acquisition of drug-tolerant phenotypes in tumorigenic cells compared to normal or immortal cells. It also suggests a remarkable sensing of microenvironmental cues in the AXL-expressing malignant cells (150). Indeed, AXL is regulated by microenvironmental and extracellular matrix components that negatively (e.g., laminin-111 and type IV collagen) or positively (e.g., type I collagen, osteopontin, IL-8, and type VIα3 collagen) impact its expression in non-malignant and malignant cell lines. Cytokines released by stromal cells such as IL-3, or GM-CSF, and thrombopoietin (TPO) can augment AXL expression in AML cells through STAT5-dependent activation (98). TGF-ß exposure upregulates AXL expression and induces a mesenchymal-like phenotype in normal and immortalized human mammary epithelial cells, breast cancer (BCa) cells, and BCa cancer stem cells (48, 121, 151). Downregulation of AXL following treatment with siRNAs or the multi-targeted tyrosine kinase inhibitor MP470 (amuvatinib) prevented this phenotypic switch, suggesting a role for AXL in TGF-ß-induced EMT (151). A plethora of EMT-aTFs regulated by TGF-ß signaling may upregulate AXL expression in various cancer systems (56, 113). Lastly, AXL was shown to be involved in murine mammary gland homeostasis, and repopulation of the ductal tree upon transplantation to cleared fat pads (150, 152).

Taken together, AXL contribute to epithelial plasticity programs in mammary stem and progenitor cells, and, when co-opted, maintains acquired stemness in BCa cells. It is well documented that AXL is particularly important for persister cells (119). Shaffer and colleagues demonstrated that human melanoma cells exhibit profound transcriptional variability at the single-cell level. Several resistance markers, including AXL, were expressed at high levels in a tiny percentage of cells, referred to as pre-existing «jackpot» cells, representing the cells that later became enriched in resistant disease (135). In sum, the data published so far point to a critical role for AXL in mediating normal and cancer cell plasticity in various contexts.

## 7 Evidence for AXL-Mediated TIME Remodeling, Immunosuppression, and Impact of Targeting AXL on Response to IMMUNE CHECKPOINT INHIBITION (ICI) in Preclinical Models

### 7.1 Influence of AXL on Immune Cell Functions

**Figure 3:**
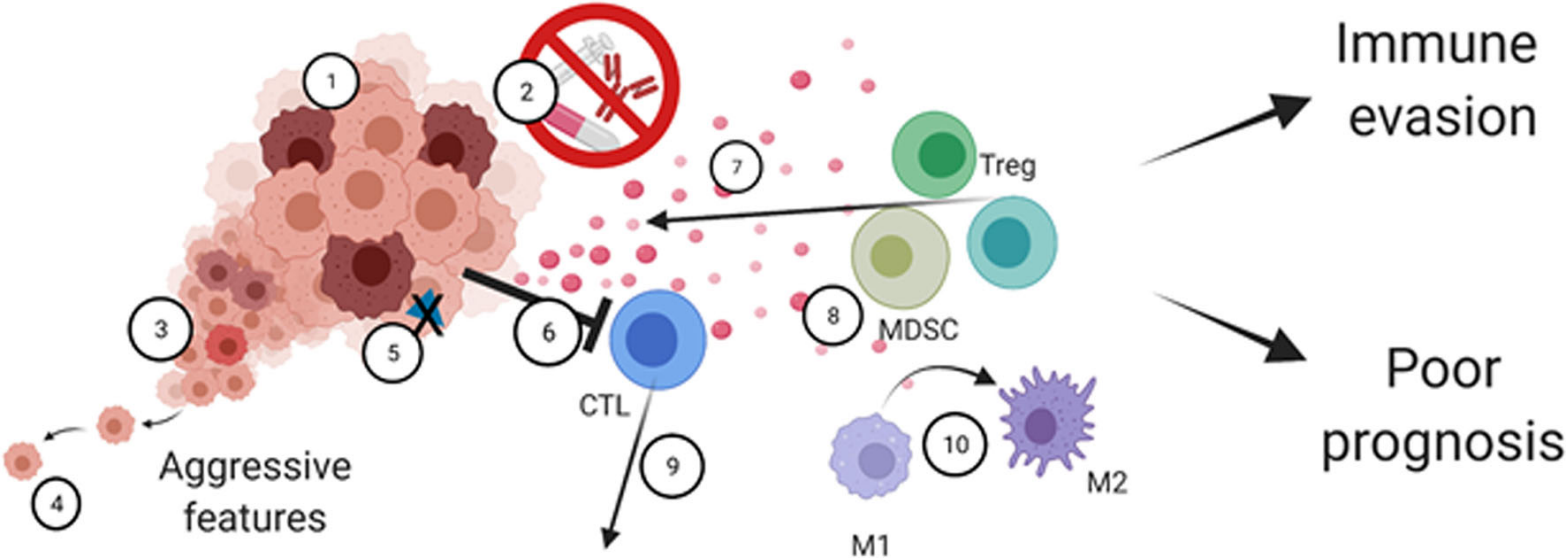
The multifaceted roles of AXL in the tumor-immune microenvironment. AXL signaling regulates cancer cell-intrinsic properties such as 1) Tumor cell growth and survival, 2) therapy resistance, 3) cancer cell plasticity mediating cancer heterogeneity and 4) increased cell motility. AXL can also mediate cancer cell immune escape through 5) decreased antigen presentation and by 6) resisting immune cell killing. AXL also mediates remodeling of the tumor microenvironment by 7) secretion of immunosuppressive cytokines and chemoattractants, 8) recruitment of immunosuppressive cells, including MDSCs and Tregs, 9) decreased infiltration of activated immune cells including cytotoxic T-cells, and 10) M1 to M2 polarization. Ultimately, this leads to tumor immune evasion and poor prognosis.

AXL can be expressed by various cells in the TME, including immune cells, fibroblasts, endothelial cells, and platelets (6, 28, 153–155). Thus, AXL should not be generalized or categorized as a tumor-specific marker. The GAS6/AXL signaling pathway strongly modulates the TME (30, 156) (**Figure 3****)**. Fibroblasts, tumor-associated macrophages, and endothelial cells are among the primary sources of GAS6 within the TME (157–160). Not to mention that malignant cells can educate non-cancer cells to produce the ligand GAS6 (66, 161). TAM receptors, including AXL, operate as essential regulators of the innate immune response. Found on the surface of various immune cells, including monocytes and phagocytic cells (162, 163), DCs (164, 165), and NK cells (166–169), they are generally recognized as having inhibitory functions on activity and/or maturation of these cell types (169), making the GAS6/AXL and TAM pathways attractive targets for therapy (170–172). As mentioned, under physiological conditions, AXL on phagocytic cells can exert essential functions in apoptotic cell clearance *via* binding the “eat-me” signal phosphatidylserine (PS) and triggering PS-mediated efferocytosis. In cancer, the TAM receptors, including AXL, promote macrophage polarization towards an immunosuppressive pro-tumor M2-like phenotype (156, 173, 174). In response to efferocytosis, macrophages are further polarized to an M2-like phenotype and secrete increased levels of immunosuppressive cytokines. Of note, the expression of TYRO3, AXL, and MERTK, as well as their ligands, can be dramatically upregulated on immunosuppressive MDSCs in *Braf-V600E/Pten* deficient melanoma tumor-bearing C57BL/6 mice (175). The suppressive capacity of MDSCs was affected in the different TAM receptor knockout mice bearing syngeneic *Braf-V600E/Pten* tumors, or after treatment with a pan-TAM inhibitor, UNC4241. This latter condition also increased CD8+ T-cell infiltration and potentiated anti-programmed cell death protein 1 (PD-1) efficacy. In another study, AXL was found to affect programmed cell death ligand 1 (PD-L1) expression in mature DCs (mregDCs) exhibiting reduced DC immunostimulatory- and enriched immunoregulatory functions (176).

### 7.2 Targeting AXL Reveals Its Role in Regulating the TIME Composition and Response to ICI

The study by Aguilera and colleagues first reported that *Axl* knockout in the murine *MMTV-PyMT* breast cancer model perturbed tumor growth while increasing tumor radiosensitivity (177). Most strikingly, loss of *Axl* was associated with multiple changes in the TIME composition, including a more significant proportion of antigen-presenting myeloid DCs (CD11c+, Major Histocompatibility Complex (MHC) class II+) and an increase in CD8+ T cell infiltration. These changes correlated with decreased secretion of myeloid supportive cytokines (*Csf1*, encoding macrophage colony-stimulating factor 1, *Csf2*, encoding *g*ranulocyte-macrophage colony-stimulating factor, and *Csf3*), chemoattractants (*Ccl3*, *Ccl4*, and *Ccl5*), and NF-kappaB target cytokines (*Il6*, *Tnfa*, and *IL1a*). Anti-PD-1 or anti-PD-1/CTLA-4 treatment alone was not sufficient to achieve tumor regression in this model, possibly due to insufficient priming of T cells by myeloid CD206-DCs and/or the absence of dominant tumor antigens in this system. However, the combination of radiotherapy with this immunotherapy regimen resulted in tumor regressions.

A recent study using the *Her2+* mouse model of BCa provides clues to understand the complex molecular network mediated by AXL (178). Using conditional deletion approaches, the investigators found that AXL expression in tumors enhances hypoxia in the TIME associated with poor vascularization, which impacts the activity and composition of immune cells. AXL creates the optimal conditions for both immunosuppression and metastatic progression. In agreement with the above study, *Axl* KO cells secreted lower amounts of CCL2, CSF1, CXCL1, and CXCL2, but also altered the secretome of other cell types such as tumor-associated CD206-macrophages, which were shown to produce less VEGFA, CXCL1, and CXCL2. *Axl* KO tumors were found to be significantly enriched for cytotoxic NK cells, B cells, and pro-inflammatory macrophage, suggestive of improved immune responses compared to *Axl*+/+ derived CD206-tumors. Conversely, immunosuppressive Tregs and pro-tumoral CD206+ macrophages were less abundant in *Axl* KO compared to *Axl*+/+ tumors. Another notable finding is that AXL could promote a hypoxic state in breast carcinoma cells by stabilizing tumoral HIF-1α through AXL-HER2 interaction (178). This cooperative event greatly contributed to shaping an immunosuppressed TIME. Furthermore, *Axl* deletion in these mouse models led to a more antitumorigenic TIME and enhanced anti-PD-1 efficacy with significant responses observed against primary and metastatic lesions. An elegant work using two immunocompetent syngeneic murine models of BCa (4T1 and E0771) has investigated how differential expression of AXL expression on tumor cells and MERTK on immune host cells could cooperate to promote immune evasion and immunosuppression (179). Targeting *Axl* through genetic means and MERTK by antibodies significantly reduced tumor growth, metastatic spread, and improved survival of immunocompetent mice when combined with anti-PD-1. The effects appeared to be dependent on T cell infiltration/activation following treatments. Interestingly, targeting MERTK in immune-host cells proved superior over *Axl* KO to increase the quality of the anti-tumor immune infiltrates when administered with anti-PD-1. In particular, a net increase in the number of cytotoxic T and NK cells was noted specifically under anti-MERTK plus anti-PD-1 treatment (179).

Other studies have primarily used pharmacological approaches to define the therapeutic potential of AXL targeting. In mice bearing tumors from the ovarian mouse ID8 line, Guo et al., showed that treatment with the selective AXL inhibitor, bemcentinib, improved survival outcomes of the animals (180). This treatment led to an accumulation of tumor-infiltrating effector CD4+ and CD8+ T cells and CD206-CD103+ cross-presenting DCs. Minor effects were observed on other immune populations such as Tregs, monocytes/macrophages, conventional DCs, and NK cells. The investigators also noted a significant decrease in immunosuppressive substances such as *Arginase 1 (Arg1)*, *Tgf-b1*, *Il-10*, and monocytic/macrophage chemo-attractants (*Ccl2/3/4*), and *Ccl5*, but an increase in pro-inflammatory chemo-attractants (*Cxcl-9/10/11* and *Cxcl-12*) (180). Further analysis indicated that AXL inhibition upregulated expression of PD-L1 and MHC I (on murine tumor cells) and PD-1 (on T cell populations) and that AXL inhibition, using bemcentinib or SGI-7079, combined with PD-1 blockade resulted in cure of ID8 and 4T1 tumor-bearing mice. Upregulation of PD-L1 and MHC-I molecules in treated tumors was consistent with the induction of an adaptive immune response, coinciding with the production of IFN-γ, which could potentiate anti-PD-1 effects. The work of Jia et al. identified a novel GAS6/AXL regulatory pathway in the triple-negative breast cancer (TNBC) cell line, MDA-MB-231, in which GAS6 expression is upregulated by FBXO7 overexpression controlling SIX1/EYA2-mediated transcriptional activation of GAS6, and mesenchymal features of the cells (181). In human MDA-MB-231 tumor-bearing mice, treatment of the mice with an EYA2 inhibitor, namely MLS000544460, demonstrated anti-tumor effects. In contrast, in the syngeneic 4T1 TNBC model, this effect was limited, but anti-PD-1 efficacy was potentiated, and associated with tumor growth delays, increased NK and CD8 infiltrates, including IFN-γ secreting cell populations.

Other convincing studies exploiting distinct immunocompetent mouse tumor models have provided further evidence for a causal role of AXL in mounting an immunosuppressive TIME (182–184). A recent study found that mice bearing KPL (*Stk11/Kras/Trp53* mutants) lung tumors are refractory to anti-PD-1 (184). Bemcentinib treatment resulted in significant re-sensitization to anti-PD-1, and correlated with increased TCF1-expressing CD8 T cells. Comparable effects were observed in humanized mice transplanted with human NSCLC cell lines harboring *KRAS* and *STK11* mutations (i.e., A549, H2122). In the syngeneic KPL model, AXL inhibitor acts primarily on DCs, rather than on carcinoma cells or macrophages, promoting DC-mediated type I interferon secretion, infiltration of the TCF1+PD-1+CD8 cells, and response to anti-PD-1. The tumor specimens of three NSCLC patients [participants of an ongoing clinical trial (NCT03184571)] with *STK11* mutations were analyzed. Consistent with the preclinical findings, these patients showed stable disease and partial response to the pembrolizumab/bemcentinib combination.

AXL inhibition in the *Kras/cdKn2a* mutated model (KIC) of pancreatic cancer was accompanied by a reduction in IL-7, CCL11, IL6, and IL-1ß levels, as well as a net reduction in F4/80+ tumor-associated macrophages expressing ARG1, a potent immunosuppressive enzyme (182). The proportion of monocytic MDSCs (CD11b+Ly6G- Ly6C+) positive for PD-L1 also appears to be decreased. In syngeneic murine glioblastoma models, targeting AXL plus PD-1 effectively prolonged the survival of glioblastoma-bearing mice (183). A recent study has reported similar results using a new selective AXL/FLT3 inhibitor, SKI-G-801 in B16F10 melanoma, CT26 colon, and 4T1 BCa models (185). The results highlight the potential of AXL targeting to overcome anti-PD-1 therapy resistance. In the study of Ireland and colleagues, the blockade of GAS6 using warfarin in pancreatic models further revealed that inhibition of the GAS6/AXL axis could reduce cancer cell plasticity, activate NK cells and inhibit pancreatic cancer metastasis (157).

Taken together, these studies performed in numerous models have identified potential mechanisms of AXL-mediated immunosuppression, such as decreased tumor antigen presentation, suppression of pro-inflammatory cytokines, and disruption of immune infiltrates (**Figure 3**). Mechanistically, despite some similarities in the reported cytokine profiles, it is important to note that the immune cells linked to observed effects may differ between studies due to tumor model-dependent context, or differences in experimental design. In summary, the GAS6/AXL signaling may promote macrophage, monocyte, and MDSC infiltration, decrease tumor abundance of mature DCs, NK CD4+ and CD8+ T-cells. AXL targeting is frequently associated with better response to anti-PD-1 in various syngeneic mouse tumor models.

## 8 Evidence for AXL-Mediated Cancer-Cell Intrinsic Mechanisms of Immune Evasion: Further Rationale for AXL Targeting to Enhance Anti-Tumor Immune Responses

### 8.1 AXL and PD-L1

**Figure 4:**
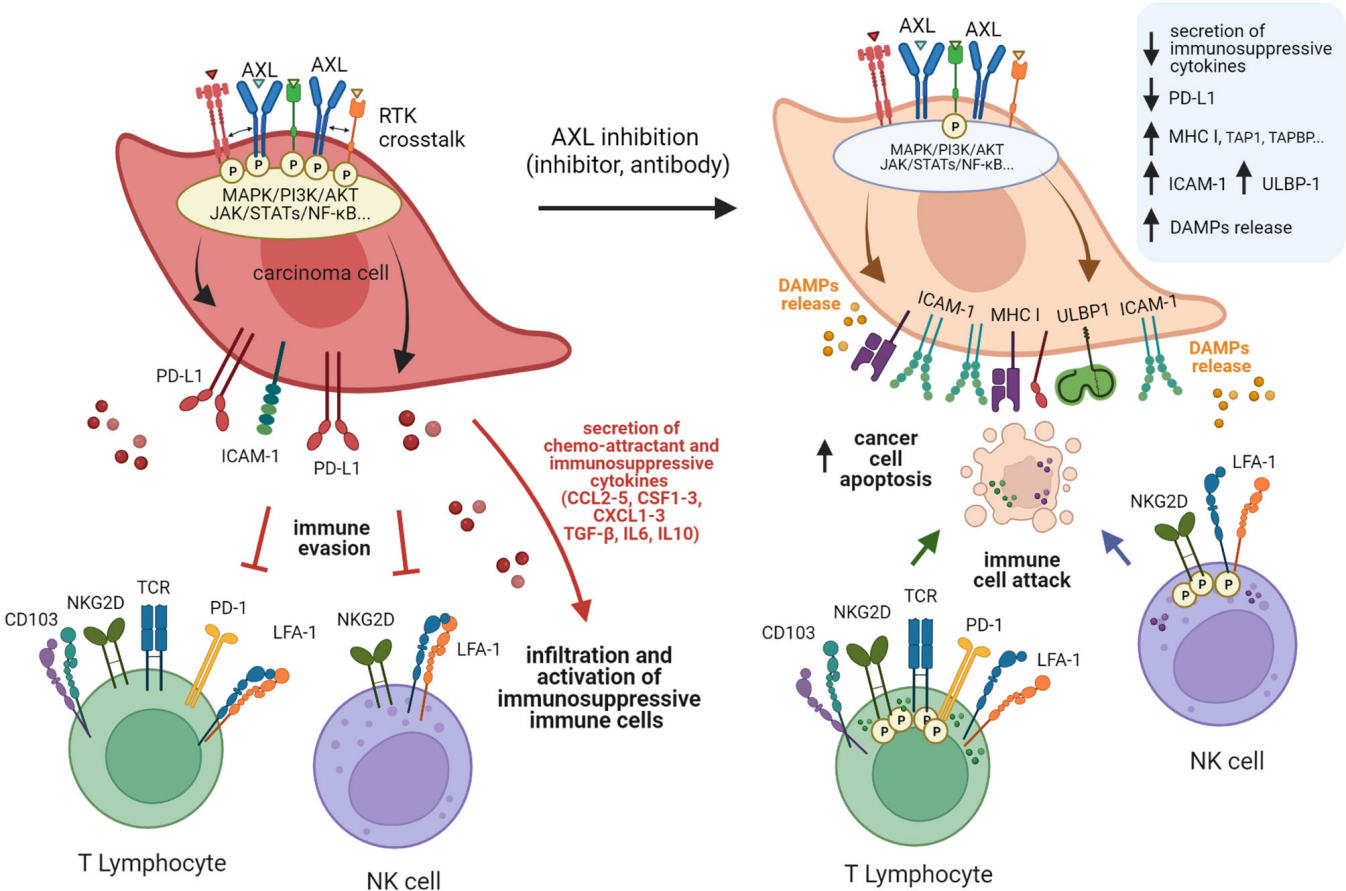
Schematic model of AXL-mediated mechanisms of immune escape and the various facets of immunosenzitisation induced by targeting AXL. High AXL expression endows cancer cells with the ability to evade immune-mediated recognition and killing through multiple mechanisms. Cancer cells with active AXL signaling generally express more PD-L1 but less MHC class I molecules, ICAM-1, and NKG2D ligands than cells with inactive or reduced AXL signaling. These characteristics are associated with reduced recognition and elimination by cytotoxic lymphocytes. These cells also secrete an array of cytokines that attract immunosuppressive cell populations or directly inhibit cytotoxic immune cells, further limiting immune responses. Targeting AXL may partially reverse this phenomenon and sensitize carcinoma cells to immune attacks, while amplifying immune responses through the induction of immunogenic cell death.

GAS6/AXL signaling may not simply act as a regulator of immune stromal cells in the TIME. Recent studies also suggest that TAM receptors, such as MERTK and AXL may contribute to immunosuppression through cancer intrinsic mechanisms (**Figures 3**, **4**), such as increased PD-L1 expression on tumor cells (186). PD-L1 and PD-L2 can prevent T cells from killing tumor target cells through binding to cell surface PD-1 expressed by T lymphocytes, thereby attenuating the immune response. Under certain conditions, a functional PD-L1/PD-1 axis may serve to predict response to anti-PD-1 therapy. However, generalizations should be avoided, mainly because PD-L1 expression can be controlled by oncogenic events that drive multiple tumor escape mechanisms. In human carcinoma cells MDA-MB-231, HeLa, and MCF7, PD-L1 expression was shown to be upregulated by hyperactive MERTK and AXL signaling. Activation was potentiated by the presence of PS-presenting apoptotic cells or PS-derived vesicles in a manner that is partly dependent on PI3K/AKT signaling (186). In their study, Boshuizen et al. investigated human melanoma (BLM, SkMel-147) and lung cancer (LCLC-103H) preclinical models and found PD-L1 to be highly expressed in all tumors expressing AXL (187). In the study by Sadahiro et al., tumor expression profiling of murine glioblastoma tumors indicated that upon AXL inhibition, *Cd274* (PD-L1) was downregulated, whereas *Pdcd1lg2* (PD-L2*)* was upregulated (183). Another interesting observation is that PD-L1 was found to be expressed predominantly by tumor (CD45-) and myeloid (CD45+/CD11b+) cell subsets. However, it remains to be defined in this case if AXL targeting impacts PD-L1 expression on myeloid or CD45- cells.

In the PyMT BCa model, AXL expressing tumors displayed increased PD-L1 on tumor cells. Recently, immunohistochemistry (IHC) labeling of AXL in a series of more than 300 clear cell RCC (ccRCC) tissues revealed that high AXL expression is associated with increased expression of PD-L1 on carcinoma cells (188). In another study, analysis of mRNA profiles in NSCLC datasets indicated a correlation between *AXL* and *CD274* (PD-L1)*, CXCR4*, and *CXCR6* expressions (189). AXL/PD-L1 association was most pronounced in lung adenocarcinomas with an EGFR-mutated status, known to be more refractory to anti-PD-1 blockade (190). Furthermore, *in vitro* treatment of PC9 and H1975 cells with the AXL inhibitor bemcentinib inhibited *PD-L1*, *PD-L2*, and *CXCR6* expression in these cells (189). Collectively, these observations provide consistent clues supporting an association between AXL expression and PD-L1 to hinder antitumor immune responses (**Figure 4**).

### 8.2 AXL and MHC Class I Expression

The report by Aguilera and colleagues showed that loss of AXL in the PyMT BCa model did not only result in altered TIME composition but also in augmented levels of mouse H-2Kb MHC class I (MHC I) (177) (**Figure 4**). Cancer cells typically express tumor-specific antigens or neoantigens presented as epitopes of 8 to 11 amino acids on MHC I at the cell surface. Upon cell-cell interactions with CD8+ T cells and binding of MHC I/epitope complexes to T-cell receptors (TCR), CTL activity is promoted, triggering cell-mediated lysis by the perforin/granzyme pathway. Alternative lysis mechanisms include TNF- FAS- and TRAIL-mediated apoptosis pathways. Thus, in cases of partial or loss of expression of MHC I, defects in antigen expression or antigen processing are expected to favor tumor immune escape. In recent work, we found an association between lower amounts of MHC I and high AXL expression in lung carcinoma cell clones (113). However, AXL inhibition did not result in the upregulation of MHC I in this setting. Yet, we noted an increase in the levels of genes involved in antigen processing and presentation, such as *TAP1*, *TAPBP*, and ERAP2.

The molecular link between AXL and MHC I expression previously identified by Aguilera and colleagues in murine models was highlighted in a recent study analyzing 94 melanoma tumors collected at baseline and PD-1 inhibitor progression (191). This study further reveals that MHC I downregulation is a hallmark of PD-1 inhibitor resistance-associated with enrichment of MITF^low^/AXL^high^ dedifferentiated cancer cell populations. In addition to demonstrating the AXL/MHC I association in human tumors, the investigators reported associations with SNAIL upregulation and cancer-associated fibroblast signatures. As observed in preclinical studies, TGF-ß could promote the expansion of AXL^high^ tumor cells and interfere with MHC I expression at baseline or upon exposure to interferon-γ (IFN-γ), which in turn may favor immune evasion. These results are consistent with previous data in prostate (192) and lung cancer cells suggesting that TGF-ß is a potent repressor of MHC I expression (193), and that blocking TGF-ß signaling could be beneficial in overcoming this immunosuppressive roadblock and enhancing immune responses.

### 8.3 AXL, Damage-Associated Molecular Patterns and Immunogenic Cell Death

In another cancer model, we have shown that bemcentinib abrogates autophagic flux in erlotinib-resistant lung cancer HCC827 cells, in association with increased cell surface expression of MHC I molecules, and markers of immunogenic cell death (ICD) revealed by the release of ATP, in addition to two other damage-associated molecular patterns (DAMPs); release of High Mobility Group Box 1 (HMGB1) and cell surface-exposed calreticulin (126) (**Figure 4**). ICD has been shown to promote immune cell infiltration mediated by the DAMPs released from dying tumor cells. The DAMPs may aid in attracting antigen-presenting cells to the tumor bed, eliciting a prominent immune response (194). These results thus highlight another potential advantage of targeting AXL-expressing cancer cells, namely to warm up immune-cold tumors and thereby increase the benefit of ICI. Interestingly, the AXL-targeting agent, Enapotamab vedotin (EnaV), which is an antibody-drug conjugate (ADC), was found to stimulate the release of DAMPs in human models of lung cancer and melanoma (187). Boshuizen and colleagues demonstrated that EnaV treatment could induce markers of ICD and inflammatory responses *in vitro* and *in vivo* (187), while promoting the expression of specific immune checkpoints on cytotoxic T cells (PD-1, CD137). Most importantly, EnaV was found to enhance tumor-specific CD8+ T-cell immune responses in multiple instances and potentiate anti-PD-1 efficacy in human tumor xenograft and mice with a humanized immune system.

### 8.4 Other Mechanisms Involved in AXL-Mediated Immune Evasion

Previous work on human NSCLC carcinoma cells from the IGR-Heu model, also provided an exciting insight into AXL-mediated molecular mechanisms of tumor immune evasion (113). Among the cancer clones displaying mesenchymal phenotypes, those with high AXL expression exhibited pronounced intrinsic resistance to NK and CTL-mediated lysis. AXL targeting using bemcentinib reduced their protective advantage (113). Of note, AXL inhibition using bemcentinib could only partially restore tumor cell sensitivity to lymphocyte-mediated lysis, suggesting that additional factors mediate immune resistance and evasion of these carcinoma cells. Mechanistic studies revealed immune sensitization following AXL targeting involves a complex molecular network stimulating NF-kappaB pathway with a concomitant increase in ICAM-1 expression on the one hand, and on the other hand, inhibiting MAPK coinciding with upregulation of ULBP1 (113). Intriguingly, bemcentinib did not appear to uniformly affect the EMT program in this condition, suggesting that the observed immune sensitization of tumor cells relied on alterations in AXL signaling, rather than solely on alterations of the mesenchymal phenotype in this model. ICAM-1 on target cells binds to its cognate receptor LFA-1 (*ITGAL/ITGB2*) on effector lymphocytes (e.g., CTLs and NK cells), strengthening the interaction between the cytotoxic killer cells and carcinoma target cells (**Figure 4**). Similarly, ULBP1 binds to its cognate receptor NKG2D (KLRK1) on effector lymphocytes, enhancing the cytolytic activity of cytotoxic killer cells towards carcinoma target cells. Furthermore, mRNA expression of ICAM-1/LFA-1 and ULBP1/NKG2D has been associated with improved survival in NSCLC datasets suggesting an attractive prognostic value for these immune-related genes (113). Together, these studies have led to a better understanding of the mechanisms linking AXL, immune evasion, and immunogenicity in cancer. The role of AXL and its dynamic expression remains to be studied in depth in most malignancies. Another important challenge will be to integrate multicellular components and physiologically relevant physico-chemical parameters (e.g., hypoxia, pH, stiffness), as well as heterogeneity of the TIME into this research.

## 9 Toward Standardization of AXL-Targeting Agents in Combination With Cancer Immunotherapy: Dream or Reality?

Due to the pro-tumoral, pro-metastatic, and treatment resistance roles of AXL, numerous therapeutic interventions targeting AXL have been designed and investigated. Several investigators have covered this topic well to which the readers can be referred (7, 21, 22, 30, 36–38, 53, 119, 172, 195).

As discussed, there is now compelling preclinical evidence denoting the potential of targeting AXL to mediate sensitization of cancer cells to immune cell-mediated attack and diminish immunosuppression within the TIME, with beneficial additive or synergistic effects in combination with ICI. This potential has yet to be evaluated directly in clinical trials in most instances. Several ongoing or recruiting studies aim to assess the value of combining AXL targeting with ICI. While results are still pending in most cases, we are just at the beginning of an exciting period of discoveries leading towards better treatments for patients, even with difficult-to-treat cancers. Here, we discuss the promising potential for AXL targeting in immuno-oncology. Therapeutic targeting of AXL is possible *via* a multitude of strategies. First, it may be relevant to target the ligand GAS6 or its maturation. Secondly, the receptor itself can be targeted by small-molecule kinase inhibitors or AXL targeting antibodies.

### 9.1 Selective Targeting of AXL or GAS6

**AVB-S6-500** (Aravive/Stanford University) is a soluble receptor against GAS6 (196, 197). AVB-S6-500 is undergoing Phase I and II clinical trials in combination with avelumab (anti-PD-L1) in patients with urothelial carcinoma (phase II, NCT04004442), or with durvalumab (anti-PD-L1) in platinum-resistant and recurrent ovarian cancers (phase I/II, NCT04019288). Interestingly, a phase II study (NCT04300140) is planned to assess the safety and efficacy of AVB-S6-500 as monotherapy, or in combination with cabozantinib (a potent RTK inhibitor including AXL), or cabozantinib/nivolumab (anti-PD-1), in patients with advanced and metastatic ccRCC (**Table 1**)

**Table 1:** Summary of agents and clinical trials evaluating AXL-targeting drugs with ICI, or as CAR-T therapy.

Drug	Main Target(s)	Clinical Trial No	Phase	Cancer Type	Combination/Monotherapy	Status
**Bemcentinib (BGB324, R428)**	AXL	NCT03184558	II	TNBC	+pembrolizumab	Terminated
		NCT02872259	Ib/II	metastatic melanoma	±pembrolizumab; +dabrafenib and trametinib	Recruiting
		NCT03184571	II	NSCLC	+pembrolizumab	Recruiting
		NCT03654833	II	mesothelioma	+pembrolizumab vs atezolizumab/bevacizumab vs abemaciclib vs rucaparib vs dostarlimab/niraparib	Recruiting
**AVB-S6-500 (Batiraxcept)**	GAS6	NCT04019288	I/II	platinum-resistant or recurrent ovarian, fallopian tube or primary peritoneal cancer	+durvalumab	Active
		NCT04004442	II	urothelial Carcinoma	+avelumab	Recruiting
		NCT04300140	I/II	advanced or metastatic RCC	±cabozantinib or +cabozantinib/nivolumab	Recruiting
**Dubermatinib (TP-0903)**	AXL, ALK, Aurora-A/B, MERTK, FLT3	NCT02729298	I	refractory/recurrent NSCLC, melanomas, colorectal and ovarian carcinoma, pretreated or treated with immunotherapy or TKI	include groups ± immunotherapy or TKI	Active
**BA3011 (CAB-AXL-ADC)**	AXL	NCT03425279	I/II	advanced solid tumours (NSCLC, pancreatic cancer, melanoma, ewing sarcoma, osteosarcoma, leiomyosarcoma, synovial sarcoma, liposarcoma, Soft tissue sarcoma, bone sarcoma, refractory sarcoma)	±nivolumab	Recruiting
		NCT04681131	II	NSCLC	±PD-1 inhibitor	Recruiting
		NCT04918186	II	ovarian cancer	+durvalumab	Recruiting
		NCT03425279	II	soft tissue and bone sarcomas	±PD-1 inhibitor	Recruiting
**ONO-7475**	AXL, MER, TYRO3, FLT3, PDGFRα, TRKA/B	NCT03730337	I	advanced solid tumours	±nivolumab (ONO-4538)	Recruiting
**Q702**	AXL, MER, CSF1R	NA	NA	esophageal, gastric, hepatocellular, and cervical cancers	+pembrolizumab	Designed
**CCT301-38 (CAR-T)**	AXL	NCT03393936	I/II	recurrent or refractory stage IV RCC	monotherapy	Active
		NCT05128786	I	relapsed or refractory AXL positive sarcomas	monotherapy	Recruiting
**AXL-anti-PD1/CTLA4-scFv-PD1 KO-CAR-T) (among ohers)**	AXL	NCT03198052	I	lung cancer	-/+ CART to PSCA, MUC1, HER2, Mesothelin, GPC3 EGFR, or B7-H3	Recruiting
**AXL-CAR-T (among ohers)**	AXL	NCT04842812	I	advanced and recurrent cancers	monotherapy	Recruiting
**Sitravatinib (MGCD516)**	AXL, MER, MET, KIT, TRKA/B, DDR2, VEGFR1/2/3, EPHA3, TYRO3	NCT03575598	I	HNSCC, SCC of mouth, and oral cavity	+nivolumab (neoadjuvant)	Completed
		NCT02954991	II	metastatic NSCLC	+nivolumab	Active
		NCT03941873	I/II	HCC, gastric/gastroesophageal cancer	±tislelizumab	Active
		NCT03680521	II	ccRCC (locally-advanced)	+nivolumab (neoadjuvant)	Active
		NCT04887870	III	advanced or metastatic solid malignancies	±nivolumab, pembrolizumab, enfortumab vedotin, ipilimumab	Recruiting
		NCT03906071 (SAPPHIRE)	III	NSCLC (metastatic non-squamous)	+nivolumab vs docetaxel	Recruiting
		NCT04921358	III	recurrent NSCLC	+tislelizumab vs docetaxel	Recruiting
		NCT04518046	I	metastatic ccRCC and solid tumors	+nivolumab/ipilimumab	Recruiting
		NCT04727996	II	advanced biliary tract cancer	+tislelizumab	Recruiting
		NCT03606174	II	urothelial carcinoma	+nivolumab, +pembrolizumab/enfortumab Vedotin	Recruiting
		NCT05104801	II	unresectable or metastatic melanoma	±tislelizumab	Recruiting
		NCT03170960	I/II	locally advanced or metastatic solid tumors	+atezolizumab	Recruiting
		NCT04925986	II	NSCLC (non-squamous, advanced treatment-naïve PD-L1+)	+pembrolizumab	Not yet recruiting
		NCT04904302	II	metastatic or advanced ccRCC	+nivolumab	Not yet recruiting
		NCT04734262	II	recurrent or metastatic TNBC	+tislelizumab	Not yet recruiting
		NCT05228496	II	small cell lung cancer	+tislelizumab	Not yet recruiting
**Cabozantinib**	VEGFR2, AXL, MET, KIT, RET, FLT-3, TIE-2	NCT03141177 (Checkmate 9ER)	III	advanced ccRCC	+nivolumab vs sunitinib alone as first line	Active
		NCT03937219 (COSMIC-313)	III	advanced ccRCC	+nivolumab/ipilimumab vs nivolumab/ipilimumab	Active
		NCT04338269 (CONTACT-03)	III	recurrent ccRCC or nccRCC after ICI	±atezolizumab	Active
		NCT03468985	II	advanced NSCLC	±nivolumab vs ±nivolumab/ipilimumab vs nivolumab	Active
		NCT04471428 (CONTACT-01)	III	recurrent NSCLC after anti-PD-L1/PD-1 and chemotherapy	+atezolizumab vs docetaxel	Active
		NCT03299946	I	HCC	+nivolumab (neoadjuvant)	Active
		NCT01658878 (CheckMate040)	I/II	HCC	+nivolumab vs +nivolumab/ipilimumab vs nivolumab	Active
		NCT02496208	I	metastatic genitourinary Tumors	+nivolumab/ipilimumab	Active
		NCT03316586	II	cabozantinib for metastatic TNBC	+nivolumab	Completed
		NCT03793166 (PDIGREE)	III	recurrent RCC after Nivo/IPI	±nivolumab as second line	Recruiting
		NCT04322955 (Cyto-KIK)	II	ccRCC	+nivolumab (neoadjuvant)	Recruiting
		NCT03635892	II	advanced nccRCC	±nivolumab	Recruiting
		NCT03755791 (COSMIC-312)	III	HCC	±atezolizumab vs sorafenib as first-line	Recruiting
		NCT03539822	II	advanced gastroesophageal and gastrointestinal malignancies	+durvalumab or +durvalumab/tremelimumab (anti-CTLA4)	Recruiting
		NCT05007613	II	recurrent or metastatic esophageal SCC	+atezolizumab	Recruiting
		NCT04963283	II	refractory metastatic colorectal cancer	+nivolumab	Recruiting
		NCT04446117 (CONTACT-02)	III	metastatic castration-resistant prostate cancer (mCRPC)	+atezolizumab vs abiraterone/enzalutamide/prednisone	Recruiting
		NCT04400474	II	endocrine and neuroendocrine tumors	+atezolizumab	Recruiting
		NCT04477512	I	metastatic hormone sensitive prostate cancer	+nivolumab/Abiraterone	Recruiting
		NCT03866382	II	rare genitourinary tumors	+nivolumab/ipilimumab	Recruiting
		NCT04289779	II	muscle-Invasive bladder cancer	+atezolizumab (neoadjuvant)	Recruiting
		NCT03824691	II	advanced and chemotherapy-treated bladder carcinoma	+durvalumab	Recruiting
		NCT04514484	I	advanced cancers with HIV	+nivolumab	Recruiting
		NCT04230954	II	recurrent and metastatic cervical cancer	+pembrolizumab	Recruiting
		NCT03824691	II	advanced and chemotherapy-treated bladder carcinoma	+durvalumab	Recruiting
		NCT04820179	II	recurrent and metastatic pancreatic cancer	+atezolizumab	Not yet recruiting
		NCT05092958	III	metastatic urothelial cancers	+avelumab vs avelumab maintenance	Not yet recruiting
		NCT05039281	II	recurrent glioblastoma	+atezolizumab	Not yet recruiting
		NCT05019703	II	recurrent or metastatic osteosarcoma	+atezolizumab	Not yet recruiting
		NCT05111574	II	mucosal melanoma	+nivolumab (adjuvant)	Not yet recruiting

Information was obtained from www.clinicaltrials.gov. Abbreviations: ccRCC, clear cell renal cell carcinoma; nccRCC, non-clear cell renal cell carcinoma; RCC, renal cell carcinoma; NSCLC, non-small cell lung cancer; TNBC, triple-negative breast cancer; HCC, Hepatocellular carcinoma; HNSCC, Head and neck squamous cell carcinomas; SCC, Squamous Cell Carcinoma.

**Bemcentinib/BGB324/R428** (BerGenBio ASA/Rigel Pharmaceuticals) is being tested for different indications. Bemcentinib is a type 1 inhibitor showing preferential specificity for AXL compared to MERTK and TYRO3, and high potency to inhibit both ligand-dependent and ligand-independent AXL signaling. As mentioned above, it has been used by many investigators in preclinical studies to demonstrate the roles of AXL in tumorigenesis, EMT, metastatic spread, therapy resistance, and immunosuppression (48, 51, 56, 151, 180, 198, 199). A single-arm study has evaluated the combination of bemcentinib with pembrolizumab (anti-PD-1) as second-line treatment in NSCLC (phase II, NCT03184571) (**Table 1**). In metastatic melanoma, another study will assess bemcentinib in combination with pembrolizumab or dabrafenib/trametinib (phase I/II NCT02872259). In Mesothelioma, bemcentinib/pembrolizumab is being compared to other targeted therapies (phase I/II, NCT03654833). Assessment of bemcentinib/pembrolizumab effects in refractory TNBC patients (phase II, NCT03184558) was discontinued as none of the participants achieved a complete or partial response (n=29). Likewise, a study combining cabozantinib with nivolumab failed to demonstrate sufficient efficacy in TNBC (phase II, NCT03316586). Therefore, the most lethal form of breast cancer remains a clinical challenge, despite converging preclinical evidence demonstrating the efficacy of AXL/TAM inhibitors combined with PD-1 blockade in TNBC models. As pembrolizumab plus neoadjuvant chemotherapy has recently been established as standard of care for TNBC patients (200), the combination of bemcentinib with the standard of care chemo-immunotherapy regimen would be of great interest. Targeting alternative checkpoints (e.g., CD47, TIM3, CTLA4, adenosine receptors 2) could provide benefits to emerging therapies targeting the PI3K, AKT, androgen receptor, CDK4/6 or PARP (201).

**Dubermatinib/TP-0903** (Tolero Pharmaceuticals/Sumitomo Dainippon Pharma) is another reported selective inhibitor of AXL that may have additional targets such as ALK, Aurora-A, and -B, FLT3, and MERTK (202–204). Compared to bemcentinib, it has a more potent cytotoxicity (130, 205). While it is interesting for specific indications such as in AML, to our knowledge, its clinical value in combination with immunotherapy remains to be investigated. In phase I clinical trial in patients with advanced solid tumors (NCT02729298), dubermatinib was given to heavily pretreated patients as a single agent or combined with immunotherapy or a TKI. Subgroup analysis may be informative about the potential efficacy of this agent.

**BA3011/CAB-AXL-ADC** (BioAtla) is an antibody-drug conjugate (ADC) consisting of an AXL-targeting antibody conjugated to an undisclosed cytotoxic agent. The clinical safety and efficacy of BA3011, alone or in combination with PD-(L)1 blockade, is being evaluated in clinical studies involving patients with NSCLC (Phase II, NCT04681131), ovarian cancers (Phase II, NCT04918186), solid tumors (Phase I NCT03425279), and soft tissue and bone sarcomas (Phase II, NCT03425279).

The use of antibody-drug conjugates is considered a promising strategy in the context of tumor heterogeneity. Using anti-AXL receptor antibodies as vectors to deliver cytotoxic agents to the tumor would elicit a cytotoxic agent in target-expressing cancer subsets. It could also have anti-cancer effects through bystander killing capacity on surrounding cancer cell subsets, with low or no target antigen expression.

**HuMax-AXL-ADC**/Enapotamab Vedotin/AXL-107-MMAE (Genmab) is another ADC incorporating a potent anti-cancer microtubule-targeting agent, Monomethyl auristatin E (MMAE) (206). Preclinical data demonstrated interesting properties of this ADC (187). HuMax-AXL-ADC has been evaluated in Phase I/II clinical study (NCT02988817) for patients with advanced solid tumors of various types. However, Genmab will not advance the development of this agent. The company has announced some evidence of clinical activity, and preliminary results have not reached proof-of-concept (company announcement, Nov 24, 2020 at 4:40 PM CET, https://ir.genmab com)

**AXL-CAR-T** cells have entered clinical trials (phase I for lung and solid tumors NCT03198052; NCT04842812, phase I/II refractory stage IV RCC NCT03393936, and sarcomas NCT05128786). Chimeric antigen receptor (CAR) T cells directed to AXL emerged as an attractive immunotherapeutic approach from preclinical studies (207, 208). Results of various CAR-T studies for the treatment of solid tumors have been less impressive in terms of efficacy compared to that observed in hematological cancers (209). Nevertheless, CAR technology, engineering, and knowledge of CAR-T biology have expanded rapidly in recent years. CAR-based therapies may offer new therapeutic solutions in the management of patients with lethal diseases. One significant remaining challenge is the tumor heterogeneity and tumor-specificity of targeted antigens. Because AXL expression is not restricted to malignant cells, AXL-CAR-T studies must be carefully monitored for potentially significant side effects, with a rigorous analysis of the AXL expression profile.

Collectively, these studies will confirm or refute AXL as a unique and targetable marker of metastasis, therapy resistance, and immune evasion while providing information on the safety, tolerability, and efficacy of the combinations relative to therapeutic standards. It should be noted that there is evidence, as shown by the example of dubermatinib, that some inhibitors previously considered selective for AXL may be potent against other targets and depending on the context (202–204).

### 9.2 Multi-Kinase Inhibitors With a Proven Effect on AXL

There is a growing body of preclinical and clinical evidence suggesting that approaches targeting multiple TAM or AXL and other RTK receptors are particularly well suited to potentiate the efficacy of ICI. Such agents are attractive due to their potential to act on a variety of independent mechanisms, thereby reducing the likelihood of resistance. However, in some cases, these drugs may suffer from increased toxicity. Some compounds with a broader range of targets than just AXL are considered.

#### 9.2.1 Multi-Target Inhibitors Selective for TAMs in Preclinical/Early-Stage Clinical Development

**RXDX-106** (Ignyta) is a selective TAM (TYRO3, AXL, MERTK) inhibitor that can also inhibit MET and RON kinases (210, 211). RXDX-106 entered Phase 1 evaluation, but the trial was terminated (NCT03454243). In the preclinical setting, RXDX-106 had significant antitumor activity in multiple syngeneic tumor models (MC38, Renca, EMT-6) acting both on the tumor and immune cell compartments (210). RXDX-106 administered alone was associated with tumor growth delays and a significant increase in tumor-infiltrating leukocytes, M1-polarized intratumoral macrophages, and activation of NK cells, indicative for mobilization and activation of both innate and adaptive anti-tumor immunity in treated mice (210). RXDX-106 exhibited effects *via* direct actions on TAM receptors expressed on intratumoral macrophages, DCs, and tumor cells. Indirect effects were also perceived on macrophages, DCs, NK cells, CD4+ and CD8+ T cells. Interestingly, the investigators noted variations in AXL and MERTK expression during tumor progression and treatment exposure. In two murine colorectal tumor models commonly used in the immuno-oncology field (CT26 and MC38), RXDX-106 further potentiated the effects of anti-PD-1 therapy, correlating with enhanced antitumor efficacy and survival of mice (210). Clinical trials are warranted to confirm the clinical relevance of the findings. As claimed by the investigators, RXDX-106 may have a more durable target engagement compared to other agents that are more advanced in their clinical development (e.g., bemcentinib or cabozantinib). More work is needed to investigate this intriguing possibility in appropriate models. Additionally, it would be interesting to know if this holds true against other anti-TAM receptor compounds.

**INCB081776** (Incyte) is a novel AXL/MERTK inhibitor that might also inhibit MET. A phase 1 study will explore the safety and tolerability of INCB081776 in patients with advanced malignancies (INCB081776). In mice bearing established MC38 and 4T1 tumors, it showed interesting additive effects with anti-PD-L1, by reducing tumor growth by 70% and 55%, respectively (212). Other agents are of interest because of their selectivity for TAMs, coupled with the fact that they are being clinically evaluated in phase 1. These include **ONO-7475** (Ono Pharmaceutical Co), selective for AXL, MER, TYRO3, and FLT3 (127), which will be evaluated in AML and solid tumors (NCT03176277 NCT03730337). **Q702** (Qurient Co) has been reported with selectivity for AXL, MERTK, and CSF1R (213). Dose escalation and safety profile are evaluated (NCT04648254). The company also announced the design of a phase 1b/2 trial evaluating the combination with pembrolizumab. **MRX-2843**/UNC2025 (Meryx, Inc) is selective for MERTK, FLT3 with less activity against AXL and TYRO-3 (214, 215). Phase 1 studies are enrolling patients with refractory/advanced NSCLC (NCT04762199) and solid tumors (NCT03510104).

#### 9.2.2 Multi-Target Inhibitors for AXL, TAM, and Non-TAM RTKs

**Sitravatinib**/MGCD516 (Mirati Therapeutics) is a multitargeted kinase inhibitor that potently enhances the efficacy of anti-PD-1, as observed in the murine KLN205 lung- as well as in the murine E0771 breast- cancer models (216). Sitravatinib targets VEGFR1/2/3, c-KIT, MET, DDR2, and TAM receptors (TYRO3, AXL, MERTK). *In vitro*, the investigators used mouse bone marrow-derived macrophages to show that sitravatinib can prevent monocytes from polarizing into immunosuppressive macrophages in cultures containing immunosuppressive conditioned medium and IL-4. Importantly, *MerTK*–/– BMDMs were unaffected by sitravatinib, indicating a central role for MERTK on macrophage polarization in these conditions. In tumors derived from the murine KLN205 cancer cells, sitravatinib significantly reduced the proportion of PD-L1-expressing-MDSCs and tumor-associated macrophages among CD11b+ cells and increased T-cell infiltration (CD3+, CD4+ CD8+, Ki67+ PD-1+ CTLA-4+). The authors suggest that the observed antitumor activity primarily relates to microenvironmental changes upon sitravatinib exposure. This study focused on the role of MERTK. Still, given the distinct and complex expression patterns of AXL, MERTK, and TYRO3, it would be interesting to investigate whether sitravatinib can also target AXL on the different cell subsets.

Sitravatinib has been investigated in combination with anti-PD-1 in different settings, as neoadjuvant treatment for locally advanced ccRCC (phase II, NCT03680521) with promising data. The investigators noted stable disease and partial responses but no patients with progressive disease while on treatment, suggesting the clinical activity of this combination. These results appear to correlate with an increase in tumor immune cell infiltration and inflammatory signatures (217). In the preoperative setting, the SNOW study demonstrated that sitravatinib plus nivolumab was safe and efficacy in oral cavity carcinomas (NCT03575598 (218). Another study in NSCLC is currently evaluating this combination in the metastatic setting (NCT02954991). Sitravatinib alone or combined with tislelizumab has been tested in unresectable advanced/metastatic hepatocellular carcinoma (HCC) and gastric cancer (phase I/II, NCT03941873). Other studies are planned (NCT04904302, NCT04925986, NCT04734262), or recruiting (NCT04518046, NCT04727996, NCT03606174), including three phase III studies to assess combination therapies in different settings for advanced/recurrent NSCLC (NCT03906071, NCT04887870) and various malignancies (NCT04921358).

**BMS-777607**/ASLAN002 (ASLAN Pharmaceuticals/Bristol-Myers Squibb) is a MET/RON inhibitor that also inhibits AXL and TYRO3 (219). The therapeutic potential of the pan-TAM/MET inhibitor BMS-777607 in combination with anti-PD-1 was examined in the murine breast cancer E0771 model (219). In this model, AXL expression is weak in macrophages and high in tumor and DCs, whereas MERTK and TYRO3 expression is marginal in carcinoma cells and restricted to macrophages and DCs. BMS-777607 promoted antitumor, and antimetastatic activity, as well as host antitumor responses while synergizing with anti-PD-1 therapy. In a Phase I trial (220) (NCT01721148), the drug was considered well-tolerated. It resulted in long-term stable disease and partial responses in certain tumor types, leading to initiation of a Phase II trial and results pending (NCT00605618).

**Cabozantinib**/BMS-907351/Cabometyx/XL184/Cometriq (Exelixis/Ipsen) is a potent inhibitor of VEGFR2, MET, RET, AXL, KIT, FLT3, and Tie2 (221). It is approved for various indications, including first-line and recurrent metastatic RCC and HCC. Often considered an angiogenesis inhibitor, preclinical studies have also shown its potential to inhibit EMT (222, 223). The specific effect of this drug on AXL is an interesting follow-up question that should be studied further. Additionally, cabozantinib may promote immunomodulation toward an immune-permissive TME, supporting the development of cabozantinib in combination with immunotherapy in various indications (224–226). In solid tumors, recent clinical trials revealed increased efficacy in combination with anti-PD(L)-1. The COSMIC-021 is a Phase Ib non-randomized study of cabozantinib in combination with atezolizumab in subjects with locally advanced or metastatic disease (NCT03170960). The trial is enrolling 24 cohorts in 12 tumor types and up to 1,720 patients. Initial results presented at the Asco meetings showed favorable safety and efficacy profiles for this combination in patients with various cancer types, including NSCLC, RCC, urothelial cancer, and castration-resistant prostate cancers.

In RCC, the phase III Checkmate 9ER study (NCT03141177) evaluated cabozantinib + nivolumab versus sunitinib as first-line treatment in patients with advanced ccRCC. The initial results published by Choueiri and colleagues are encouraging. With a median follow-up of 18.1 months, nivolumab plus cabozantinib demonstrated significant superiority over the antiangiogenic agent sunitinib, in terms of median progression-free survival (PFS)(16.6 months vs 8.3 months), overall survival (OS) at 12 (months 85.7% vs 75.6%) and, objective response rate (55.7% vs 27.1%) (227). Preliminary results from the recently reported phase II trial, NCT03635892, also indicate a promising safety profile and efficacy of cabozantinib + nivolumab in advanced RCC with non-clear cell histology, including papillary RCC [ORR 54% and 36% as of 1st line or 2nd line, respectively (228)]. The Phase III trial COSMIC-313 (NCT03937219) explores the safety and efficacy of the triplet therapy (cabozantinib + nivolumab + ipilimumab) versus nivolumab and ipilimumab for patients with intermediate- or poor- risk advanced RCC (229). The CONTACT-03 study (III, NCT04338269) is now active to evaluate cabozantinib alone or in combination with atezolizumab in metastatic RCC patients who experienced progression during/after ICI treatment. In the PDIGREE Study [phase III NCT03793166 (230)] after the first-line treatment with nivolumab and ipilimumab, patients with metastatic RCC will receive cabozantinib in the event of progression, nivolumab versus nivolumab cabozantinib in the event of partial response or with stable disease, while patients with complete responses (CR) will receive nivolumab as maintenance therapy. In NSCLC, a phase II study (NCT03468985) evaluates the benefit of adding cabozantinib to nivolumab, or nivolumab plus ipilimumab, versus nivolumab alone in the treatment of patients with recurrent Stage IV NSCLC. In the CONTACT-01 study (phase III, NCT04471428), atezolizumab combined with cabozantinib versus docetaxel is evaluated in patients with metastatic NSCLC previously treated with an anti-PD-L1/PD-1 and platinum-containing chemotherapy. In Hepatocellular carcinoma (HCC), the COSMIC-312 trial (Phase 3, NCT03755791) evaluates the safety and efficacy of cabozantinib plus atezolizumab versus sorafenib, or cabozantinib alone as first-line treatment for advanced disease (231). Exelixis and the investigators recently announced encouraging results with significant PFS benefits compared to single agents and a trend toward improved OS at the interim analysis (232). The final analysis of OS results is expected for early 2022. In Phase I/II trial NCT01658878, patients with advanced liver cancer (CheckMate040) receive nivolumab or nivolumab in combination with other agents, including nivolumab plus cabozantinib, and nivolumab plus ipilimumab plus cabozantinib. In patients with metastatic castration-resistant prostate cancer, a phase III study (NCT04446117, CONTACT-02) will evaluate the value of cabozantinib/atezolizumab over abiraterone/enzalutamide/prednisone treatments. Additional phase I or II studies are planned, recruiting, or ongoing to evaluate the safety and efficacy of cabozantinib-based combinations in numerous malignancies (**Table 1**).

## 10 Future Considerations in the Use of AXL/TAM Targeting Agents

Numerous companies have developed agents preferentially targeting AXL, TAM receptors, or AXL and related RTKs such as MET receptor. Some agents have already been discontinued, while others are in preclinical evaluation or in early phase clinical trials, with the potential to expand the therapeutic arsenal in the future as first- or subsequent-lines of therapy for specific indications. It might be beneficial to use those sequentially or utilize synergistic combinations to increase effectiveness against cancer, preventing the emergence of acquired resistance and compensatory mechanisms (233). With the increasing number of available agents targeting AXL, it will be essential to learn more about the extent to which these agents differentiate in terms of efficacy and toxicity. TAM receptors are expressed by many different cell types. Thus, one can assume higher toxicity for anti-TAM agents in case of high potency. It would be interesting to know if highly potent pan-TAM inhibitors can be beneficial over more selective inhibitors for AXL, GAS6, MERTK, or TYRO3. Analysis of clinical and outcome measures in separate studies can be informative but certainly not sufficient in this regard. Clinical data comparing the available agents are lacking. The fact that some completed studies have not reported their results is also problematic, raising concerns about high toxicity or low efficacy. A clinical trial design optimized with different dose schedules and incorporating predictive biomarkers may fulfill some of these requirements. Molecular markers that may assist in therapeutic decision-making are needed. Both preclinical and clinical studies should devote efforts to assess correlations between the target expression and therapeutic response. Confirmation of AXL expression or activation is not systematically verified, augmenting the risks associated with unnecessary treatment, increased morbidity, and unnecessary costs.

## 11 AXL and Related-TIME Components as Promising Biomarkers for Treatment Decisions

In a recent survey of 316 ccRCC cases, high AXL expression in tumor cells was associated with a lower response rate to anti-PD-1 therapy in metastatic ccRCC patients who were refractory to anti-angiogenic agents in the NIVOREN phase II trial (188). High AXL expression was also associated with increased PD-L1 tumoral expression, and ccRCC patients with concomitant PD-L1 and high AXL expression in their tumor specimens had the worst OS. The effect of AXL on clinical outcomes and PD-L1 expression was preferentially observed in tumors with loss of the VHL tumor suppressor, a key regulator of hypoxia through targeting HIF for proteasomal degradation under normoxia, evoking the hypoxia-dependent nature of these associations. *VHL* inactivation, however, did not seem to influence AXL mRNA or protein expression levels in this cohort of samples, suggesting that AXL action, rather than AXL expression level *per se*, is magnified by hypoxia in this setting.

Hakozaki et al. recently reported a large-scale analysis of primary and metastatic RCC lesions (234). The investigators showed that combined GAS6/AXL scoring, obtained from IHC on tissue microarray (TMA) samples, was an independent marker of poor prognosis following surgery, proving superior to each staining alone. The analyzed cohorts may differ from cohorts of patients currently treated with ICI for advanced and metastatic disease, and it is yet unproven whether this scoring may predict resistance to ICI therapy. Beyond showing that AXL combined with GAS6 immunostaining is predictive for poor prognosis, the investigators discovered novel associations between a high GAS6/AXL score and increased CD73, CD47, CSF1R, LAMP2, and IDO-1 immunostainings in primary lesions, suggestive of an immunosuppressed TIME in this subset. CD73 and LAMP2 were also strongly associated with a high GAS6/AXL profile in metastatic lesions, supporting their essential roles in the progression of these tumors. Intriguingly, among many other molecules tested, including immune checkpoint receptors (PD-1, CTLA-4, TIM3) and PD-L1, none was found to be correlated with GAS6/AXL status, except LAG3 in the metastatic setting. The lack of correlation with PD-L1 is in contrast with previous preclinical studies (183, 186, 189), as well as the recent observations of an association between tumoral expression of AXL and PD-L1 in the Nivoren RCC Cohort (188). One intriguing possibility is that GAS6 may interfere with the AXL-PD-L1 association. It would be interesting to investigate further the potential relationships between AXL, GAS6, PD-L1, CD73 and their spatial distribution, since other investigators have found in TNBC samples a preference of AXL-expressing carcinoma cells to be in contact with the stroma (58).

Importantly, under some circumstances, hypoxia and pseudohypoxia may act on transcriptional regulation of AXL or its protein stabilization (98, 112, 113, 235). AXL was also found to be a component of the IPRES gene signature along with other known HIF targeted genes like *ROR2*, *WNT5A*, *LOXL2 VEGFA*, and *VEGFC* (236). IPRES stands for “innate PD-1 resistance signature’’ and was developed from metastatic melanoma transcriptomic data. The IPRES signature is characterized by upregulation of EMT-related factors, including AXL, immunosuppressive cytokines, hypoxia, and pro-angiogenic factors. Unfortunately, the signature was not confirmed to be associated with PD-1 inhibitor response in subsequent studies (237). In melanoma, tumors enriched for MITF^low^/AXL^high^ population seemed more likely to be resistant to PD-1 inhibition (191).

Various signatures have been proposed to predict response to ICI that have yet to be validated prospectively. This includes signatures that capture the immune response, such as the 18-gene tumor inflammation signature (TIS) that measures the expression of inflammatory genes such IFN-γ signature (238), as well as the cytolytic index (CYT), which is based on the expression of GZMA (granzyme A) and PRF1 (perforin) expression, that serve as surrogate markers of T-cell cytotoxic activity (239). In 44 pre-treated melanoma tumors, the expression of these signatures, as well as the presence of CD8+ T cell population, did not accurately predict response to anti-PD-1 (191). This could be explained in part by the finding that the inflamed/non-inflamed status may exhibit significant intratumor heterogeneity between biopsies taken before and after treatment (191). This work highlights the risk of relying solely on baseline predictive biomarkers to guide a patient’s treatment trajectory, as they may not capture inherent tumor heterogeneity, much like the selective pressures evoked by ICI modifying a tumor’s evolutionary trajectory. Thus, testing of biopsies from different tumor sites should be incorporated when validating such biomarkers, and the impact of heterogeneity on their clinical relevance should be examined. Although ethically questionable and complicated to implement in clinical practice, longitudinal sampling would be beneficial to advance our knowledge in this respect. The TIME is critical to effective therapy, and although the contribution of AXL or GAS6/AXL-related signatures in predicting ICI resistance remains unproven (133, 234), it would be of great interest to study how surrogate markers of immune response in combination with such signatures could improve their predictive value. As observed in numerous cancer systems, associations between AXL, TGF-ß signaling, and reduced levels of MHC class I deserve to be further investigated.

## 12 Concluding Remarks and Remaining Challenges

Despite significant advances in the field of cancer immunotherapy, to date, survival benefits have been limited to a minority of patients, and durable clinical responses are rare. It has become clear that the TIME plays a crucial role in mediating response to treatment. In this regard, understanding how tumors escape anti-tumor immunity and characterizing the underlying mechanisms of immunotherapy resistance is critical. AXL and other TAM receptors are now recognized to play essential roles in regulating the TIME. Accumulating evidence indicates that AXL may confer intrinsic and extrinsic capacities to avoid destruction by immune effector cells. However, the underlying mechanisms by which AXL contributes to counteracting anti-tumor defenses are not yet fully understood and merit further investigation in several malignancies. Moreover, the molecular links between tumor cell plasticity, AXL expression, and resistance to immunotherapy remain unclear. More studies are required to understand the interactions between the different cellular components of the TIME, as well as many elements affecting tumor metabolism such as hypoxia, nutrient depletion, pH deregulation, oxidative or mechanical stressors, all of which could regulate AXL expression and/or selection of AXL-expressing cells. Research is also needed to elucidate the epigenetic determinants of AXL expression.

Developing pharmacological agents to modulate AXL activity or the GAS6/AXL pathway is attracting attention in the field of immuno-oncology. These agents hold the potential to act on both cancer and stromal compartments, thus combining anti-cancer effects and stromal remodeling towards an immune permissive TIME and immune sensitization of cancer cells. We are at the dawn of a new area of discoveries based on an increasingly detailed knowledge of AXL biology, the development of compounds targeting AXL, or multiple TAM receptor family members. These compounds are being evaluated in a wide array of cancer patients either as monotherapy or in combination clinical trials with immunotherapies, mainly anti-PD-1 or anti-PD-L1. These clinical trials will hopefully lead to significantly improved patient survival, and will also contribute to a better understanding of AXL biology to set the directions for future basic and translational research efforts. Integrating biomarker studies will be crucial to guide and interpret clinical results. We and others assume that molecular testing and target evaluation on cancer and immune cells in the TME should accompany these efforts to ultimately increase the clinical response rates. Finally, novel therapy combinations incorporating other immune checkpoints (e.g., CD47, TIM3, LAG-3, TIGIT, CTLA4, adenosine receptors 2) should also be considered.

## Author Contributions

J-PT, JL, and SC: writing review and editing. AE, ML, RAK, and ST: investigation, writing original draft, writing review and editing, visualization, conceptualization. All authors contributed to the article and approved the submitted version.

## Funding

ST would like to acknowledge the support received by Association pour la Recherche sur les Tumeurs de la Prostate (ARTP), Institut National du Cancer (INCa), and Cancéropôle Ile-de-France (2021-1-EMERG63). RAK and SC would like to acknowledge the support of the Sheikh Hamdan Foundation. SC would like to acknowledge the support received by ITMO-Cancer.

## Conflict of Interest

The authors declare that the research was conducted in the absence of any commercial or financial relationships that could be construed as a potential conflict of interest.

## Publisher’s Note

All claims expressed in this article are solely those of the authors and do not necessarily represent those of their affiliated organizations, or those of the publisher, the editors and the reviewers. Any product that may be evaluated in this article, or claim that may be made by its manufacturer, is not guaranteed or endorsed by the publisher.
